# Impact of Body Mass Index on the Development of Inflammatory Bowel Disease: A Systematic Review and Dose-Response Analysis of 15.6 Million Participants

**DOI:** 10.3390/healthcare9010035

**Published:** 2021-01-03

**Authors:** Akshaya Srikanth Bhagavathula, Cain C.T. Clark, Jamal Rahmani, Vijay Kumar Chattu

**Affiliations:** 1 Department of Social and Clinical Pharmacy, Faculty of Pharmacy, Univerziti Kralova, 500 03 Hradec Kralova, Czech Republic; bhagavaa@faf.cuni.cz; 2 Centre for Intelligent Healthcare, Coventry University, Coventry CV1 5FB, UK; cain.clark@coventry.ac.uk; 3 Department of Community Nutrition, Faculty of Nutrition and Food Technology, National Nutrition and Food Technology Research Institute, Shahid Beheshti University of Medical Sciences, Tehran 198396-3113, Iran; jrahmani@sbmu.ac.ir; 4 Division of Occupational Medicine, Department of Medicine, Faculty of Medicine, University of Toronto, Toronto, ON M5G 2C4, Canada; 5 Occupational Medicine Clinic, St. Michael’s Hospital, Unity Health Toronto, Toronto, ON M5C 2C5, Canada

**Keywords:** obesity, body mass index, inflammatory bowel disease, Crohn’s disease, systematic review, meta-analysis, dose-response analysis

## Abstract

Background: A growing trove of literature describes the effect of malnutrition and underweight on the incidence of inflammatory bowel disease (IBD). However, evidence regarding the association between underweight or obesity and IBD is limited. The study aimed to assess the association of body mass index (BMI) with a risk of IBD (Crohn’s disease (CD) and ulcerative colitis (U.C.)) incidence. Methods: We systematically searched PubMed/Medline, Cochrane, Web of Science, and Scopus for observational studies assessing the association between BMI and IBD that were published up to 30 June 2020. We estimated pooled hazard ratios (HR) with corresponding 95% confidence intervals (CI). Random effect dose-response meta-analysis was performed using the variance weighted least-squares regression (VWLS) models to identify non-linear associations. Results: A total of ten studies involving 15.6 million individuals and 23,371 cases of IBD were included. Overall, obesity was associated with an increased IBD risk (HR: 1.20, 95% CI: 1.08–1.34, *I*^2^ = 0%). Compared to normal weight, underweight (BMI < 18.5 kg/m^2^) and obesity (BMI ≥ 30 kg/m^2^) were associated with a higher risk of CD, and there was no difference in the risk of U.C. among those with BMI < 18.5 kg/m^2^ and BMI ≥ 30 kg/m^2^. There was a significant non-linear association between being underweight and obesity and the risk of development of CD (Coef_1_ = −0.0902, *p*_1_ < 0.001 Coef_2_ = 0.0713, *p*_2_ < 0.001). Conclusions: Obesity increases the risk of IBD development. Underweight and obesity are independently associated with an increased risk of CD, yet there is no evident association between BMI and the risk of U.C. Further studies are needed to clarify the underlying mechanism for these findings, particularly in CD.

## 1. Introduction

Globally, there has been a precipitous rise in the incidence of inflammatory bowel disease (IBD) in newly industrialized countries, particularly in younger populations [1]. Historically, IBD has been associated with malnutrition and underweight [2]. Indeed, the major forms of IBD, Crohn’s disease (CD) and ulcerative colitis (U.C.), are enigmatic inflammatory disorders and cause progressive damage to the gastrointestinal tract, which leads to chronic underweight status [3]. Moreover, existing data suggest that around 65–75% of patients with CD and U.C. (18–62%) are underweight [4].

Toward the end of the twentieth century, obesity emerged as an adverse prognostic factor of various chronic inflammatory diseases, including IBD [5]. Current data on the role of obesity in IBD development are inconclusive; indeed, studies have shown a positive correlation between IBD and obesity, where 32.7% of IBD patients are obese (30.3% of CD and 35.2% of U.C.) [6]. Moreover, obesity is associated with perianal complications of CD, higher rates of relapse, and hospitalization [7,8], and several cohort studies have reported that obesity is associated with an increased risk of developing CD but not U.C. [9,10,11,12,13]. However, limited data are characterizing the impact of obesity on the development of U.C., suggesting that obesity may be higher or not associated with an increased risk of U.C. [14,15], although this has not been consistently reported [9,10,11,12,13,14,15,16,17].

The influence of body mass index (BMI) and the development of IBD has previously been documented in epidemiological evidence, with no evidence of the association between increasing B.M.I. and IBD development in the European Prospective Cohort Study (EPIC) [15]. Moreover, two prior meta-analyses were conducted on this topic, where the study by Dang et al. included 24 studies that identified a significant difference in the BMI of patients with IBD in the active phase [18], and Rahmani et al. reported obesity as a significant risk factor for the development of CD [19]. In contrast, a recent retrospective study [20] reported an absolute increase in the incidence of CD among middle-aged adults from 1.71% in those with BMI < 18.5 kg/m^2^ to 0.73% in BMI > 25 kg/m^2^. Considering the evolving epidemiology of IBD, identifying and controlling the modifiable risk factors offers disease prevention avenues. Thus, this systematic review and meta-analysis aimed to assess body mass index association with the risk of development of IBD (CD and U.C.).

## 2. Materials and Methods 

### 2.1. Data Source and Search Strategy

We performed a systematic review and meta-analysis following the guidelines of the Preferred Reporting Items for Systematic Review and Meta-analysis (PRISMA) [21]. A comprehensive literature search was conducted to identify the observational studies investigating the association between body mass index with IBD in the adult population, via PubMed/Medline, Cochrane Library, Web of Science, and Scopus, up to June 2020. A detailed search string using different combinations of MeSH (Medical Subjects Headings) keywords, using the Boolean operators “AND” and “OR”, was conducted to identify the studies (Supplementary Table S1).

All of the articles and relevant reviews published in English as full peer-reviewed manuscripts were screened for potential missing studies. The process was independently conducted by two reviewers (A.S.B. and J.R.), and the identified studies were evaluated for analysis suitability. After eliminating duplicates, titles and abstracts were screened to exclude the irrelevant studies. All observational cohort studies eligible for the present study were scrutinized thoroughly, and studies that met selection criteria were retrieved for further analysis.

### 2.2. Inclusion and Exclusion Criteria

Studies were eligible for inclusion according to the following criteria: (1) peer-reviewed articles published in English, with participants aged >18 years; (2) observational cohort studies evaluating the association between BMI and concerning the risk of IBD (CD and/or U.C.); (3) reported the association as odds ratio (OR), risk ratio (R.R.), or hazard ratio (HR), with corresponding 95% confidence intervals (CI) were included.

Studies were excluded if they were conducted on children or on patients with a diagnosis of other gastrointestinal disorders, did not incorporate sufficient data of OR/RR/HR to include in the meta-analysis or estimates to compute with 95% CI, and did not stratify according to BMI. Furthermore, in vitro and non-human studies, case reports, case series, editorials, letters, interventional studies, review articles, and non-peer-reviewed articles without sufficient data were also excluded.

### 2.3. Data Extraction and Quality Assessment

The information was extracted from each study, independently and blinded by A.S.B., C.C.C., and J.R., and discrepancies were discussed by all authors and resolved through a consensus. The following information was extracted: first author, year of publication, study location, study design, sample size, mean age and gender of the study participants, duration of the follow-up, confounding factors, and summary estimates with the corresponding 95% CI of IBD risk. Data from fully adjusted models were used for the meta-analysis.

The quality of each study was assessed according to the Newcastle-Ottawa quality assessment Scale (N.O.S.) [22], evaluating three factors: patient selection, comparability of study groups, and the assessment of outcomes. An aggregated score of 6.5 to 9 indicates high quality, while 0–6 designates low quality.

### 2.4. Data Synthesis and Statistical Analysis

The meta-analysis was performed using STATA software (version 16, STATA Corporation, College Station, TX, USA), using a DerSimonian and Laird multivariate random-effects model to combine the risk estimates of IBD [23]. Normal BMI was considered a reference category to investigate IBD’s risk in association with underweight and obesity. When the study did not provide the reference category’s data, the mean or median of each BMI category was used. Heterogeneity between the studies was estimated using the Cochran *Q* test (P_heterogeneity_) and *I^2^* statistics; P_heterogeneity_ ≤ 0.10 and/or *I*^2^ > 50% indicates significant heterogeneity. To examine the potential non-linear association between BMI and IBD risk, we used restricted cubic splines with three knots at the fixed percentiles of 10%, 50%, and 90% of exposure data [24]. Statistical significance was accepted at *p* < 0.05. Funnel plots, Begg’s rank correlation test, and Egger’s regression asymmetry test were used to evaluate the publication bias.

## 3. Results

### 3.1. Study Selection and Characteristics

**Figure 1:**
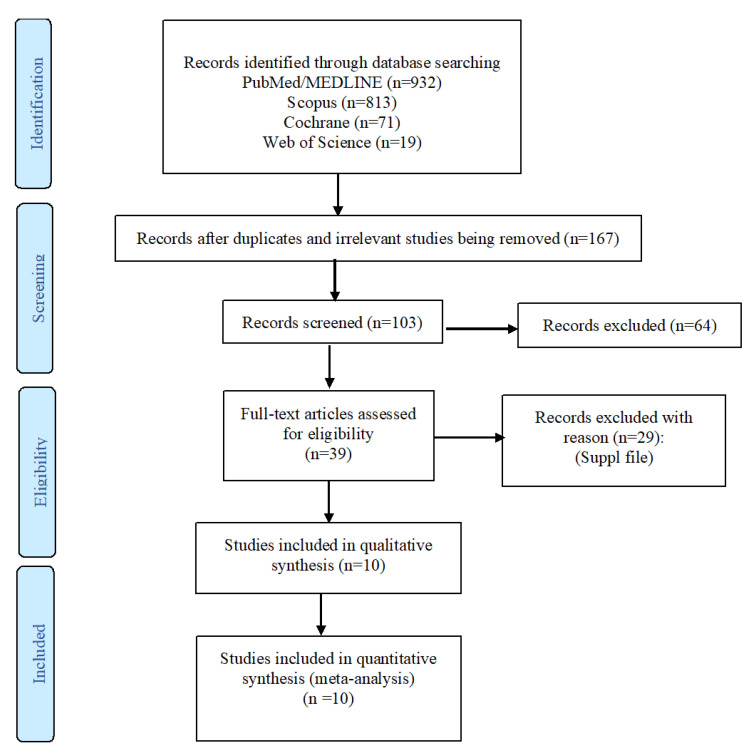
Flow chart of included studies.

The search strategy yielded a total of 1835 articles, among which 1796 were designated as not pertinent to the study purpose (Figure 1). After examining 39 full-text articles, 29 were excluded because they did not meet the inclusion criteria (Supplementary Materials). Finally, a total of 10 studies were eligible for inclusion, comprising 15,598,438 participants, and these were pooled into the meta-analysis [9,10,11,12,13,14,15,16,17,20].

The core characteristics of the included studies are presented in Table 1. Six studies were performed in Europe [9,10,11,13,15,17], two in the United States [12,16], and one each in Korea [20] and Japan [14]. The studies were published between 2013 [15] and 2020 [20]. Six studies were prospective cohorts [9,10,11,12,15,17], while four were retrospective [13,14,16,20]. Eight studies reported the cases of both CD and U.C. [9,10,11,12,13,15,17], one focused on U.C. [14], and one study reported the risk of CD in adults and the elderly [20]. The mean age of the study population was 35 years, and the median duration of follow-up was 13.4 years. Three studies were conducted on women [11,12,13], two among men [9,17], and others included participants from both genders [10,14,15,16,20]. Participants included in these studies reported a total of 23,371 cases of IBD, comprising 6097 cases of CD and 17,274 cases of U.C.

### 3.2. Primary Meta-Analysis

#### 3.2.1. Association Between BMI and Development of IBD

**Figure 2:**
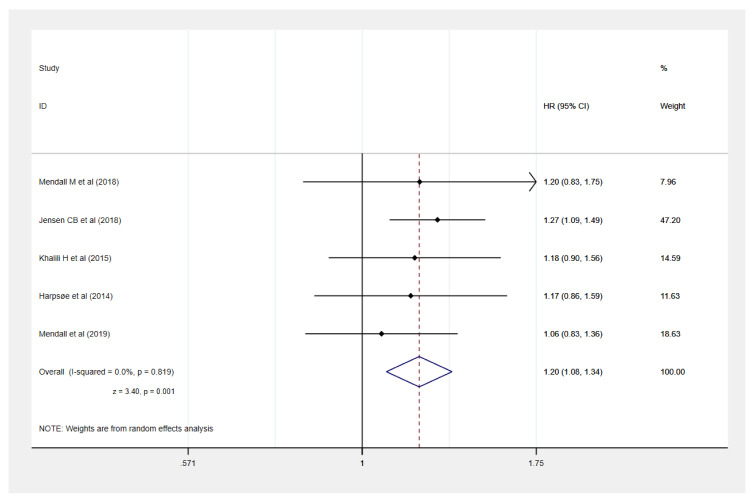
Obesity (body mass index (BMI) ≥ 30 kg/m^2^) and development of Inflammatory bowel disease.

Five studies provided the data of obesity [9,10,11,12,13] and underweight [10,11,12,17] and the development of IBD [9,10,11,12,13]. Compared to normal weight, our meta-analysis identified that obesity was significantly associated with an increased risk of IBD development by 20% (HR: 1.20, 95% CI: 1.08–1.34, *I*^2^ = 0%) (Figure 2) but not underweight (HR: 1.07, 95% CI: 0.98–1.16, *I*^2^ = 0%).

#### 3.2.2. Association Between BMI and Crohn’s Disease

**Figure 3:**
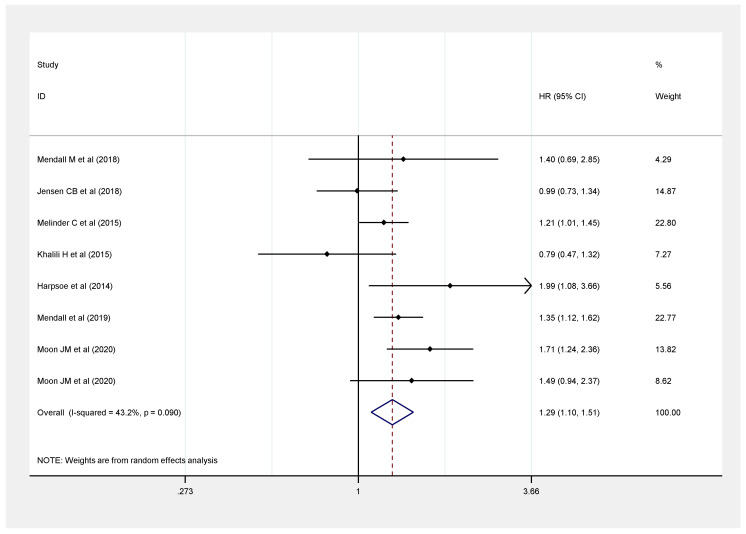
Association between underweight (BMI < 18.5 kg/m^2^) and risk of Crohn’s disease.

Seven studies provided underweight data versus the normal weight category of BMI and risk of CD from 15,257,579 participants [9,10,11,12,13,17,20]. With normal BMI as the reference, it was observed that a BMI < 18.5 kg/m^2^ poses a relatively higher risk of CD development (HR: 1.29, 95% CI: 1.10–1.51, *I*^2^ = 43.2%) (Figure 3).

**Figure 4:**
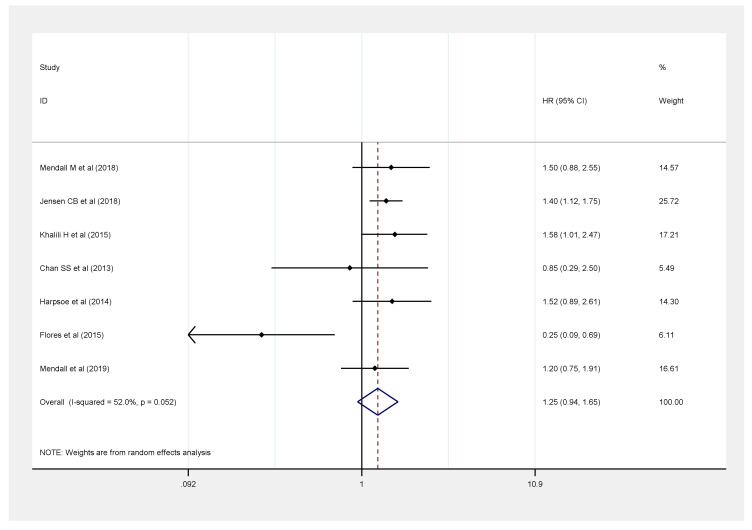
Association between obesity (BMI ≥ 30 kg/m^2^) and risk of Crohn’s disease.

There were seven studies, with 1,257,079 participants, that reported the risk of CD among obese patients [9,10,11,12,13,15,16]. A significant increase in HR was observed in those with BMI ≥ 30 kg/m^2^ compared with those with normal BMI (HR: 1.25, 95% CI: 0.94–1.65, *I*^2^ = 52%) (Figure 4).

#### 3.2.3. Association Between BMI and Ulcerative Colitis

**Figure 5:**
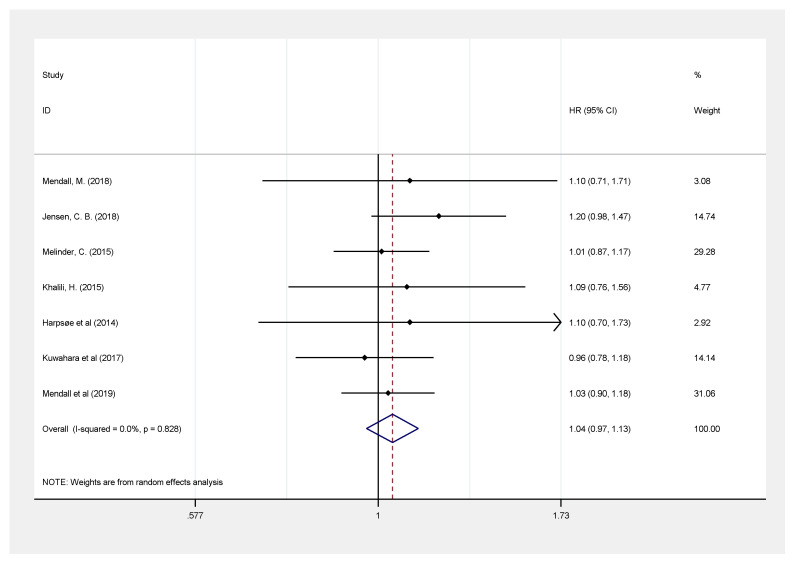
Association between underweight (BMI < 18.5 kg/m^2^) and risk of ulcerative colitis.

Seven studies including 1,236,312 participants were pooled to evaluate the risk of U.C. among patients with a BMI < 18.5 kg/m^2^ [9,10,11,12,13,14,17] and showed no significant difference in developing U.C. as compared with those with normal BMI (HR: 1.04, 95% CI: 0.97–1.13, *I*^2^ = 0.0) (Figure 5).

**Figure 6:**
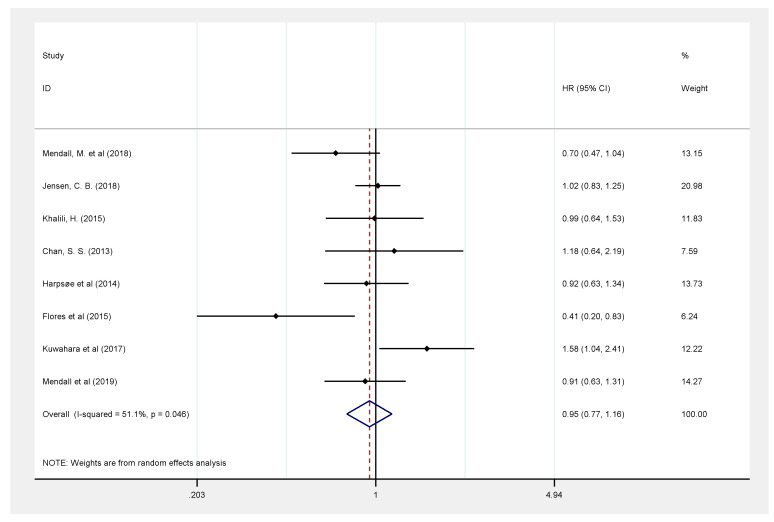
Association between obesity (BMI ≥ 30 kg/m^2^) and risk of ulcerative colitis.

Similarly, eight studies [9,10,11,12,13,14,15,16], with a total of 1,296,633 participants, reported the risk of U.C. in those with a higher BMI ≥ 30 kg/m^2^ and suggested the absence of a relationship between obesity and an increased risk of U.C. development (HR: 0.95, 95% CI: 0.77–1.16), with a significant and high heterogeneity (51.1%, *p* = 0.046) (Figure 6).

### 3.3. Dose-Response Analysis

**Figure 7:**
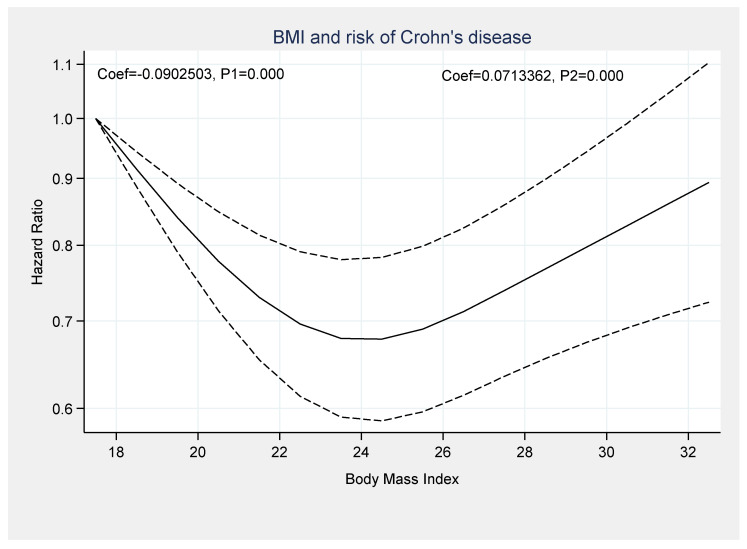
Dose-response association between BMI and development of Crohn’s disease.

A dose-response analysis was performed to assess the association between BMI and the risk of IBD. There was a significant non-linear association between being underweight or obesity and the risk of CD development (Coef_1_ = −0.0902, *p*_1_ = 0.000 and Coef_2_ = 0.0713, *p*_2_ = 0.000) (Figure 7).

**Figure 8:**
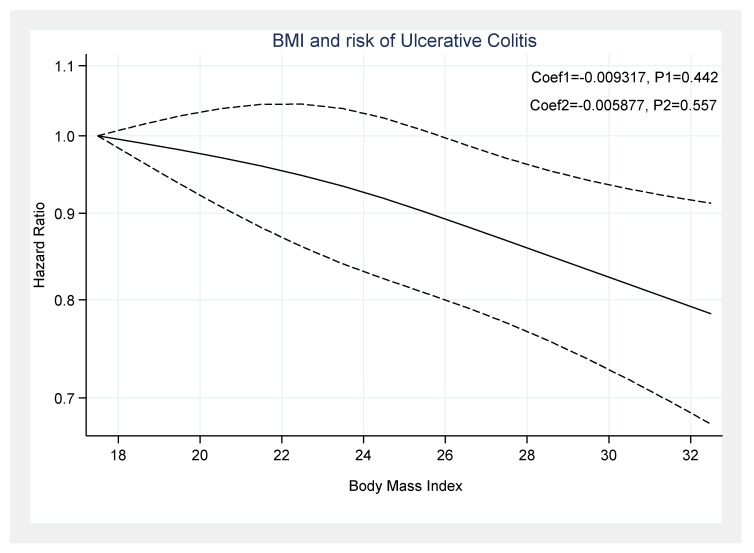
Dose-response association between BMI and development of ulcerative colitis.

However, there was no significant association between BMI and UC incidence (Coef1 = −0.0093, *p*_1_ = 0.442 and Coef_2_ = −0.0058, *p*_2_ = 0.557) (Figure 8).

### 3.4. Quality of Studies

All included studies displayed high quality, with N.O.S. scores ranging from 7 to 9. All studies were observed to have drawn the cases and controls from the same community and reported BMI. The majority of the studies provided data from a representative population and detailed the baseline and outcome exposure outcomes. Only one study did not meet the criteria for adequate assessment of the outcome of interest at baseline and did not report the baseline information [10].

### 3.5. Publication Bias

Despite the heterogeneity in the outcomes of CD and U.C., the publication bias funnel plots were found to be symmetrical. Additionally, no evidence of publication bias was identified in the primary meta-analysis in Egger’s regression asymmetry test and Begg’s rank correlation test (Supplementary Figure S1a–f).

## 4. Discussion

Worldwide, the ongoing unprecedented shift in the BMI paradigm has led to an inexorable increase in the prevalence of obesity; additionally, approximately 15–40% of IBD patients are obese [23]. On the other hand, underweight and malnutrition have been associated with IBD [24]; however, the association of underweight or obesity on the presentation and course of IBD remains equivocal. Given the increasing prevalence in the onset of IBD among young adults, understanding BMI’s association is of paramount importance. The principal findings of our meta-analysis of 10 cohort studies showed a direct association between BMI (both lower BMI and higher BMI) and risk of IBD, particularly CD. At the same time, no relationship was found between BMI and risk of U.C. Moreover, this association was further strengthened by the dose-response analysis, confirming a significant non-linear association between BMI and CD’s risk, but not U.C. This lack of association is an important message to disseminate considering the results of the present meta-analysis, which indicates that obesity may be a contributory factor in the development of CD but not U.C.

In the present study, the included articles were contemporary cohorts, all published between 2013 and 2020, which included an equitable number of prospective and retrospective studies, with an appropriately long median follow-up (≈13 years). A total of nine out of ten studies reported the association between BMI and the development of CD [9,10,11,12,13,15,16,17,20], while nine investigated the risk of U.C. [9,10,11,12,13,14,15,16,17]; one study was conducted across 10 European countries [15], one each in Japan [14], Sweden [17], and Korea [20], two in the United States [12,16], and four in Denmark [9,10,11,13].

Five studies, with a total of 879,959 individuals, were included in the dose-response analysis and reported the risk of CD and U.C. based on the BMI [9,11,12,13,17]. The results of the included studies suggested an increased risk of CD in participants with a BMI < 18.5 kg/m^2^ (HR: 1.29, 95% CI: 1.10–1.51, *I*^2^ = 43.2%) and BMI ≥ 30 kg/m^2^ (HR: 1.25, 95% CI: 0.94–1.65, *I*^2^ = 52%). A clear dose-response association was determined between BMI and CD and U.C. development in the non-linear model. Notably, we observed a paradoxical relationship in which both low and high BMI are strongly associated with a significantly increased risk of CD. Based on our analysis, there is an increased risk of CD in those with low BMI and high B.M.I., compared to normal BMI; additionally, several other studies support these results [11,13,20].

In general, obesity is linked with a chronic, low-grade, pro-inflammatory state, and it is considered an adverse prognostic factor in various chronic inflammatory diseases, including IBD [25]. While there is a range of potential pathways associated with CD development in those affected by obesity, the presence of mesenteric fat increases resistin secretion from macrophages and leukocytes. In addition, leptin secretion from adipocytes may contribute to increasing levels of inflammatory cytokines, such as tumor necrotic factor-α, and interleukin-1 and -6 [25,26,27,28,29]. Furthermore, both obesity and IBD are associated with increased gut bacterial translocation and dysbiosis [27,30]. Recently, Szilagyi’s review greatly described the pro-inflammatory pathogenic mechanism between obesity and IBD and noted specific possible interactions in both IBD and obesity [31] that can merit further investigation. A low BMI is associated with several nutritional deficiencies, micronutrients (iron, calcium, zinc, copper, magnesium, and selenium) and vitamins, such as A, D, K, B12, and folic acid, which are the main determinants for the development of IBD [4,32,33]. Thereby, both low and high BMI may introduce a two-fold challenge to the increasing IBD burden.

In this meta-analysis, both underweight and obesity were not associated with the development of U.C. This lack of association between BMI and U.C. may be due to the differences in the U.C. characteristics, such as the ulcerations being limited to the colon, the extent of mucosal damage, differences in the clinical presentation, and the extent of intestinal involvement. However, Kuwahara et al. reported an inverse association between a high BMI and the risk of U.C. exacerbation [14]. Although this putative relationship warrants further elucidation, it is important to note that just a single study reported that a high BMI is associated with an increased risk of U.C. Therefore, these results should be replicated, preferably in case-control settings, to better understand the etiology of IBD in individuals with obesity.

### Strengths and Limitations

There are several significant strengths of our meta-analysis that should be noted. Firstly, the present study has provided deeper clarity into the effect of BMI on CD and U.C. by integrating the results from 10 cohort studies, with an unprecedented, extremely large sample size, consisting of 15.6 million participants. This sample size yielded generalizable findings and the opportunity for subsequent dose-response analysis. Second, the current study was free from significant publication bias, low heterogeneity, and symmetrical funnel plots, suggesting that the study findings are reliable. A possible limitation of this study is BMI as a clinical marker; it is indeed worth considering significant confounders that may exist within the compiled studies. Lastly, it is important to note that all the included studies are observational cohorts in design. We must consider the possibility of misclassification bias. Participants in the cohort may suffer an array of additional comorbidities that may have impacted somewhat in the meta-analysis.

## 5. Conclusions

Underweight and obesity are associated with the development of CD, yet no association between BMI and the risk of U.C. was evident. The dose-response meta-analysis demonstrated that increased BMI was associated with an increased risk of CD, and the curve becomes steeper with increasing BMI. Further studies are needed to discern and clarify the underlying mechanism for these findings.

## Figures and Tables

**Table 1:** Core characteristics of studies included in the meta-analysis [9,10,11,12,13,14,15,16,17,20].

Studies	Year	Country	Baseline		Study Characteristics			IBD Development (Outcomes)		Literature Quality ^†^
			Age	Gender	Cohort Design	Follow-Up (Year)	Sample Size	CD	UC	
Moon, J. M. et al. [20]	2020	Korea	50	Both	Retrospective	7.3	14,060,821	977	-	8
Mendall, M. A. et al. [9]	2019	Denmark	19	Male	Prospective	38	377,957	1523	3323	9
Jensen, C. B. et al. [10]	2018	Denmark	46	Both	Prospective	38	316,799	1500	2732	7
Mendall, M. et al. [11]	2018	Denmark	30	Female	Prospective	6	74,512	137	448	8
Kuwahara, E. et al. [14]	2017	Japan	42	Both	Retrospective	2	39,554	-	8120	9
Flores, A. et al. [16]	2015	U.S.A.	40	Both	Retrospective	5	581	297	284	8
Melinder, C. et al. [17]	2015	Sweden	18	Male	Prospective	4	240,984	986	1878	9
Khalili, H. et al. [12]	2015	U.S.A.	34	Female	Prospective	18	111,498	153	229	8
Harpsøe, M. C. et al. [13]	2014	Denmark	30	Female	Retrospective	11	75,008	138	394	8
Chan, S. S. et al. [15]	2013	10 European countries	52	Both	Prospective	5.1	300,724	75	177	9

**^†^** Newcastle-Ottawa scale; IBD—Inflammatory bowel disease; CD—Crohn’s disease; U.C.—Ulcerative colitis.

## Data Availability

The authors confirm that the datasets analyzed during the study are available from the first author or the corresponding author upon reasonable request.

## Supplementary Materials

The following are available online at https://www.mdpi.com/2227-9032/9/1/35/s1) Table S1: Search strategy (MeSH terms), and List of studies excluded with reason. Figure S1. Funnel plots to assess publication bias ((a) Inflammatory bowel disease in obese individuals, (b) Inflammatory bowel disease in underweight individuals, (c) Crohn’s disease in underweight individuals, (d) Crohn’s disease in obese individuals, (e) Ulcerative colitis in underweights individuals, and (f) Ulcerative colitis in obese individuals).

## References

[1] Abolhassani H., Alipour V. (2020). The global, regional, and national burden of inflammatory bowel disease in 195 countries and territories, 1990–2017: A systematic analysis for the Global Burden of Disease Study 2017. Lancet Gastroenterol. Hepatol..

[2] Weimers P., Munkholm P. (2018). The natural history of IBD: Lessons learned. Curr. Teat. Options Gastroenterol..

[3] Pulley J., Todd A., Flatley C., Begun J. (2020). Malnutrition and quality of life among adult inflammatory bowel disease patients. JGH Open.

[4] Scaldaferri F., Pizzoferrato M., Lopetuso L.R., Musca T., Ingravalle F., Sicignano L.L., Mentella M., Miggiano G., Mele M.C., Gaetani E. (2017). Nutrition and IBD: Malnutrition and/or sarcopenia? A practical guide. Gastroenterol. Res. Pract..

[5] Seminerio J.L., Koutroubakis I.E., Ramos-Rivers C., Hashash J.G., Dudekula A., Regueiro M., Baidoo L., Barrie A., Swoger J., Schwartz M. (2015). Impact of obesity on the management and clinical course of patients with inflammatory bowel disease. Inflamm. Bowel Dis..

[6] Bryant R.V., Trott M.J., Bartholomeusz F.D., Andrews J.M. (2013). Systematic review: Body composition in adults with inflammatory bowel disease. Aliment. Pharmacol. Ther..

[7] Steed H., Walsh S., Reynolds N. (2009). A brief report of the epidemiology of obesity in the inflammatory bowel disease population of Tayside, Scotland. Obes. Facts.

[8] Singh S., Khera R., Sandborn W.J. (2016). Obesity Is Associated with Worse Outcomes in Hospitalized Patients with Inflammatory Bowel Diseases: A Nationwide Analysis: 591. Am. J. Gastroenterol..

[9] Mendall M.A., Jensen C.B., Sørensen T.I., Ängquist L.H., Jess T. (2019). Body mass index in young men and risk of inflammatory bowel disease through adult life: A population-based Danish cohort study. Sci. Rep..

[10] Jensen C.B., Ängquist L.H., Mendall M.A., Sørensen T.I., Baker J.L., Jess T. (2018). Childhood body mass index and risk of inflammatory bowel disease in adulthood: A population-based cohort study. Am. J. Gastroenterol..

[11] Mendall M., Harpsøe M.C., Kumar D., Andersson M., Jess T. (2018). Relation of body mass index to risk of developing inflammatory bowel disease amongst women in the Danish National Birth Cohort. PLoS ONE.

[12] Khalili H., Ananthakrishnan A.N., Konijeti G.G., Higuchi L.M., Fuchs C.S., Richter J.M., Chan A.T. (2015). Measures of obesity and risk of Crohn’s disease and ulcerative colitis. Inflamm. Bowel Dis..

[13] Harpsøe M.C., Basit S., Andersson M., Nielsen N.M., Frisch M., Wohlfahrt J., Nohr E.A., Linneberg A., Jess T. (2014). Body mass index and risk of autoimmune diseases: A study within the Danish National Birth Cohort. Int. J. Epidemiol..

[14] Kuwahara E., Murakami Y., Nakamura T., Inoue N., Nagahori M., Matsui T., Watanabe M., Suzuki Y., Nishiwaki Y. (2017). Factors associated with exacerbation of newly diagnosed mild ulcerative colitis based on a nationwide registry in Japan. J. Gastroenterol..

[15] Chan S.S., Luben R., Olsen A., Tjonneland A., Kaaks R., Teucher B., Lindgren S., Grip O., Key T., Crowe F.L. (2013). Body Mass Index and the Risk for Crohn’s Disease and Ulcerative Colitis: Data from a European Prospective Cohort Study (The IBD in EPIC Study). Am. J. Gastroenterol..

[16] Flores A., Burstein E., Cipher D.J., Feagins L.A. (2015). Obesity in inflammatory bowel disease: A marker of less severe disease. Dig. Dis. Sci..

[17] Melinder C., Hiyoshi A., Hussein O., Halfvarson J., Ekbom A., Montgomery S. (2015). Physical fitness in adolescence and subsequent inflammatory bowel disease risk. Clin. Transl. Gastroenterol..

[18] Dong J., Chen Y., Tang Y., Xu F., Yu C., Li Y., Pankaj P., Dai N. (2015). Body mass index is associated with inflammatory bowel disease: A systematic review and meta-analysis. PLoS ONE.

[19] Rahmani J., Kord-Varkaneh H., Hekmatdoost A., Thompson J., Clark C., Salehisahlabadi A., Day A.S., Jacobson K. (2019). Body mass index and risk of inflammatory bowel disease: A systematic review and dose-response meta-analysis of cohort studies of over a million participants. Obes. Rev..

[20] Moon J.M., Kang E.A., Han K., Hong S.W., Soh H., Park S., Lee J., Lee H.J., Im J.P., Kim J.S. (2020). Trends and risk factors of elderly-onset Crohn’s disease: A nationwide cohort study. World J. Gastroenterol..

[21] Liberati A., Altman D.G., Tetzlaff J., Mulrow C., Gøtzsche P.C., Ioannidis J.P., Clarke M., Devereaux P.J., Kleijnen J., Moher D. (2009). The PRISMA statement for reporting systematic reviews and meta-analyses of studies that evaluate healthcare interventions: Explanation and elaboration. Br. Med. J..

[22] Stang A. (2010). Critical evaluation of the Newcastle-Ottawa scale for the assessment of the quality of nonrandomized studies in meta-analyses. Eur. J. Epidemiol..

[23] Jackson D., White I.R., Thompson S.G. (2010). Extending DerSimonian and Laird’s methodology to perform multivariate random effects meta-analyses. Stat. Med..

[24] Orsini N., Li R., Wolk A., Khudyakov P., Spiegelman D. (2012). Meta-Analysis for linear and non-linear dose-response relations: Examples, an evaluation of approximations, and software. Am. J. Epidemiol..

[25] Singh S., Dulai P.S., Zarrinpar A., Ramamoorthy S., Sandborn W.J. (2016). Obesity in IBD: Epidemiology, pathogenesis, disease course and treatment outcomes. Nat. Rev. Gastroenterol. Hepatol..

[26] Karmiris K., Koutroubakis I., Xidakis C., Polychronaki M., Voudouri T., Kouroumalis E.A. (2006). Circulating levels of leptin, adiponectin, resistin, and ghrelin in inflammatory bowel disease. Inflamm. Bowel Dis..

[27] Peyrin-Biroulet L., Gonzales F., Dubuquoy L., Rousseaux C., Dubuquoy C., Decourcelle C., Saudemont A., Tachon M., Beclin E., Odou M.-F. (2012). Mesenteric fat as a source of C reactive protein and as a target for bacterial translocation in Crohn’s disease. Gut.

[28] Konrad A., Lehrke M., Schachinger V., Seibold F., Stark R., Ochsenkuhn T., Parhofer K.G., Goke B., Broedl U.C. (2007). Resistin is an inflammatory marker of inflammatory bowel disease in humans. Eur. J. Gastroenterol. Hepatol..

[29] Bilski J., Mazur-Bialy A., Wojcik D., Surmiak M., Magierowski M., Sliwowski Z., Pajdo R., Kwiecien S., Danielak A., Ptak-Belowska A. (2019). Role of obesity, mesenteric adipose tissue, and adipokines in inflammatory bowel diseases. Biomolecules.

[30] Kim A. (2015). Dysbiosis: A review highlighting obesity and inflammatory bowel disease. J. Clin. Gastroenterol..

[31] Szilagyi A. (2020). Relationship(s) between obesity and inflammatory bowel diseases: Possible intertwined pathogenic mechanisms. Clin. J. Gastroenterol..

[32] Spooren C.E., Wintjens D.S., de Jong M.J., van der Meulen-de A.E., Romberg-Camps M.J., Becx M.C., Maljaars J.P., van Bodegraven A.A., Mahmmod N., Markus T. (2019). Risk of impaired nutritional status and flare occurrence in IBD outpatients. Dig. Liver Dis..

[33] Ciocîrlan M., Ciocîrlan M., Iacob R., Tanțău A., Gheorghe L., Gheorghe C., Dobru D., Constantinescu G., Cijevschi C., Trifan A. (2019). Malnutrition Prevalence in Newly Diagnosed Patients with Inflammatory Bowel Disease-Data from the National Romanian Database. J. Gastrointestin. Liver Dis..

