# Supplementary material for: Impact of Body Mass Index on the Development of Inflammatory Bowel Disease: A Systematic Review and Dose-Response Analysis of 15.6 Million Participants
Source: Healthcare (Basel). 2021 Jan 3;9(1):35. doi: 10.3390/healthcare9010035 (PMC7824000; doi:10.3390/healthcare9010035)

Supplementary Table 1. Search strategy (MeSH terms)

PubMed/MEDLINE	Cochrane Library	Web of Science	Scopus
(("body mass index"[MeSH Terms] OR (("body"[All Fields] AND "mass"[All Fields]) AND "index"[All Fields])) OR "body mass index"[All Fields]) AND (((("inflammatory bowel diseases"[MeSH Terms] OR (("inflammatory"[All Fields] AND "bowel"[All Fields]) AND "diseases"[All Fields])) OR "inflammatory bowel diseases"[All Fields]) OR (("inflammatory"[All Fields] AND "bowel"[All Fields]) AND "disease"[All Fields])) OR "inflammatory bowel disease"[All Fields])<br>(("body mass index"[MeSH Terms] OR (("body"[All Fields] AND "mass"[All Fields]) AND "index"[All Fields])) OR "body mass index"[All Fields]) AND ((("colitis, ulcerative"[MeSH Terms] OR ("colitis"[All Fields] AND "ulcerative"[All Fields])) OR "ulcerative colitis"[All Fields]) OR ("ulcerative"[All Fields] AND "colitis"[All Fields]))<br>(((((("Colitis, Ulcerative"[Mesh] OR "ulcerative colitis"[tiab]) OR "Inflammatory Bowel Diseases"[Mesh]) OR "Inflammatory Bowel Diseases"[tiab]) OR "Crohn Disease"[Mesh]) OR "Crohn Disease"[tiab]) OR IBD[tiab] AND (((prospective[Title/Abstract] OR longitudinal[Title/Abstract]) OR follow-up[Title/Abstract]) OR cohort[Title/Abstract])) AND ((((("Body Mass Index"[Mesh] OR "Obesity"[Mesh]) OR "Body Mass Index"[Title/Abstract]) OR Obesity[Title/Abstract]) OR BMI[Title/Abstract]) OR "body mass indices"[Title/Abstract])	("ulcerative colitis" OR "Inflammatory Bowel Diseases" OR "Crohn Disease" OR IBD) AND (prospective OR longitudinal OR follow-up OR cohort) AND ("Body Mass Index" OR Obesity OR BMI OR "body mass indices")	TI=("ulcerative colitis" OR "Inflammatory Bowel Diseases" OR "Crohn Disease" OR IBD) AND TI=(prospective OR longitudinal OR follow-up OR cohort) AND TI=("Body Mass Index" OR Obesity OR BMI OR "body mass indices")	((TITLE-ABS-KEY ("ulcerative colitis") OR TITLE-ABS-KEY ("Inflammatory Bowel Diseases") OR TITLE-ABS-KEY ("Crohn Disease") OR TITLE-ABS-KEY (ibd))) AND ((TITLE-ABS-KEY (prospective) OR TITLE-ABS-KEY (longitudinal) OR TITLE-ABS-KEY (follow-up) OR TITLE-ABS-KEY (cohort))) AND ((TITLE-ABS-KEY ("Body Mass Index") OR TITLE-ABS-KEY (bmi) OR TITLE-ABS-KEY (obesity) OR TITLE-ABS-KEY ("body mass indices")))

List of studies excluded with reason:

1. High and low body mass index may predict severe disease course in children with inflammatory bowel disease (**Conducted on pediatric population**)
2. Associations Between Obesity and the Effectiveness of Anti–Tumor Necrosis Factor-α Agents in Inflammatory Bowel Disease Patients: A Literature Review and Meta-analysis (**Not a cohort**)
3. Body Composition Using Air Displacement Plethysmography in Children With Inflammatory Bowel Disease (**Not related to our study objective**)
4. Impact of Bariatric Surgery on the Long-term Disease Course of Inflammatory Bowel Disease (**Not related to our study objective**)
5. High body mass index is not associated with increased treatment failure in infliximab treated pediatric patients with inflammatory bowel disease (**Not related to our study objective**)
6. Post-index procedural gain in body mass index is associated with recurrent ileal pouch sinus after endoscopic or surgical therapy (**Not related to our study objective**)
7. Obesity Is More Common in Children Newly Diagnosed With Ulcerative Colitis as Compared to Those With Crohn Disease (**Conducted on pediatric population**)
8. Is Bariatric Surgery Safe and Effective in Patients with Inflammatory Bowel Disease? (**Not related to our study objective**)
9. Altered body composition profiles in young adults with childhood-onset inflammatory bowel disease (**Conducted on pediatric population**)
10. Metabolic Syndrome in Inflammatory Bowel Disease: Association with Genetic Markers of Obesity and Inflammation (**Not related to our study objective**)
11. Nutritional status and body composition in children with inflammatory bowel disease: a prospective, controlled, and longitudinal study (**Conducted on pediatric population**)
12. Obesity in pediatric inflammatory bowel disease: Prevalence and disease associations in the era of biologic therapy (**Conducted on pediatric population**)
13. Adverse effect of obesity on outcomes of children newly diagnosed with inflammatory bowel disease (**Conducted on pediatric population**)
14. Outcomes of inflammatory bowel disease surgery in obese versus non-obese patients: a meta-analysis (**Not a cohort**)
15. Neither obesity nor hepatic steatosis are associated with an increased prevalent or incident colonic dysplasia in patients with inflammatory bowel disease and concomitant primary sclerosing cholangitis (**Not related to our study objective**)
16. Role of C-reactive protein as a marker for disease course in obese patients with inflammatory bowel disease (**Not related to our study objective**)
17. Correlation of body mass index (BMI) and C-reactive protein (CRP) with inflammatory bowel disease progression in an urban, afro-caribbean population (**Not a full-length article**)
18. Nutritional habits and influence of body mass index in patients with inflammatory bowel disease (**Not related to our study objective**)
19. Adjusting for body mass index makes bioimpedance spectroscopy a viable clinical tool for bedside body composition analysis in outpatients with inflammatory bowel disease (**Not related to our study objective**)
20. Infliximab-related weight gain in patients with inflammatory bowel disease: Patterns, associations, and financial impacts (**Not related to our study objective**)
21. Longitudinal follow up of body mass index as a predictor for severe disease course in children with inflammatory bowel disease (**Conducted on pediatric population**)
22. Regional fat distribution in children with inflammatory bowel disease: Association with disease activity and phenotype (**Conducted on pediatric population**)
23. Olfactory sensitivity is associated with body mass index, polymorphism in the odor binding-protein (OBPIIa) gene and inflammatory bowel disease (**Not related to our study objective**)
24. Obesity is frequent in inflammatory disease patients and associated with higher disease activity in Crohn's disease, but not in ulcerative colitis (**Not a full-length article**)

25. High and low body mass index may predict severe disease course in children with inflammatory bowel disease (**Conducted on pediatric population**).
26. The risk of inflammatory bowel disease in subjects presenting with perianal abscess: findings from the THIN database. (**Perianal abscess patients**)
27. High body mass index is associated with increased risk of treatment failure and surgery in biologic-treated patients with ulcerative colitis. (**Not stratified BMI appropriately**)
28. Predictors of early readmission in hospitalized patients with inflammatory bowel disease. Inflammatory bowel diseases. (**Not stratified Crohn's disease and Ulcerative colitis separately**)
29. Comparing the clinical outcomes of young-onset and adult-onset ulcerative colitis: a multi-center Korean Association for the Study for Intestinal Diseases study. (**Not stratified BMI appropriately**)

Supplementary Figure 1. Funnel plots to assess publication bias

a. Inflammatory bowel disease in Obese individuals

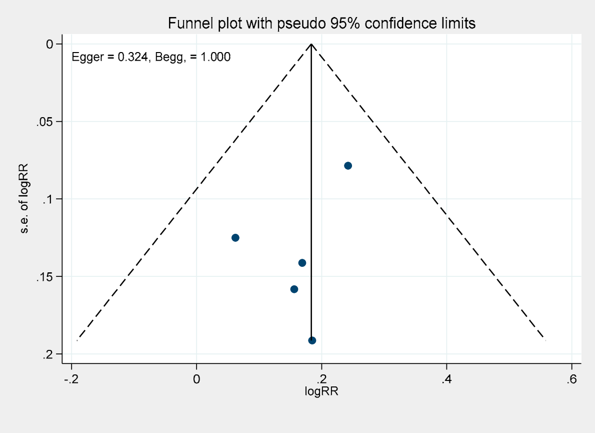

b. Inflammatory bowel disease in underweight individuals

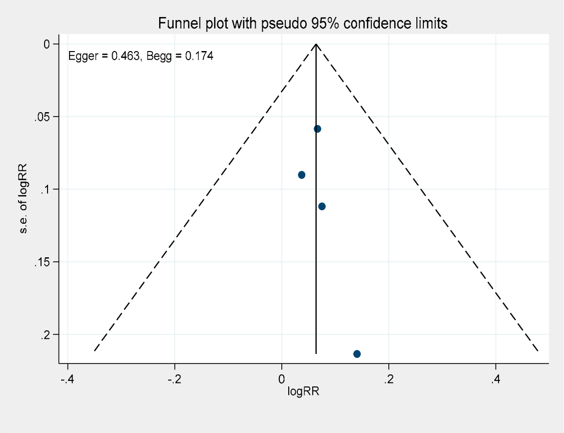

c. Crohn's disease in underweight individuals

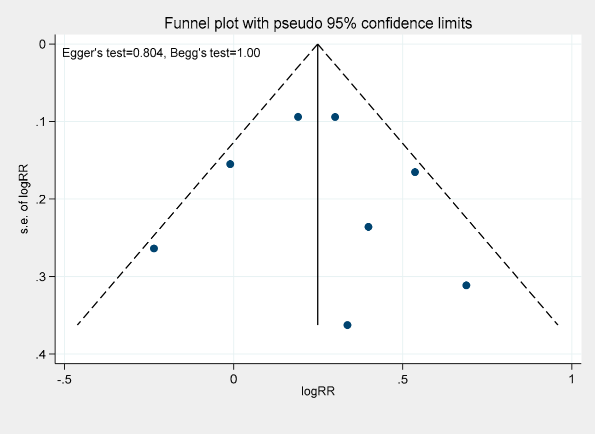

d. Crohn's disease in obese individuals

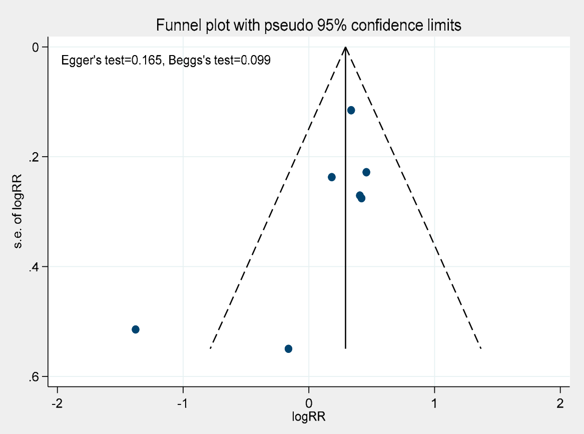

e. Ulcerative colitis in underweight individuals

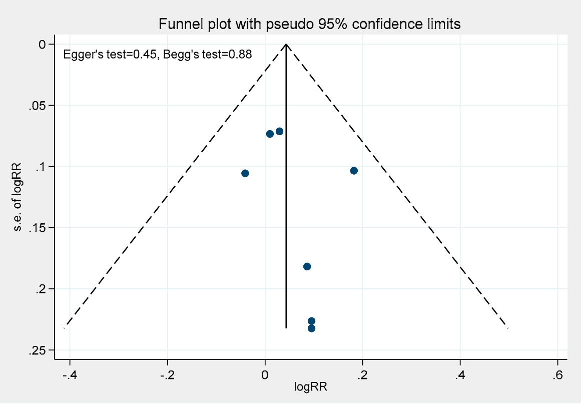

f. Ulcerative colitis in obese individuals

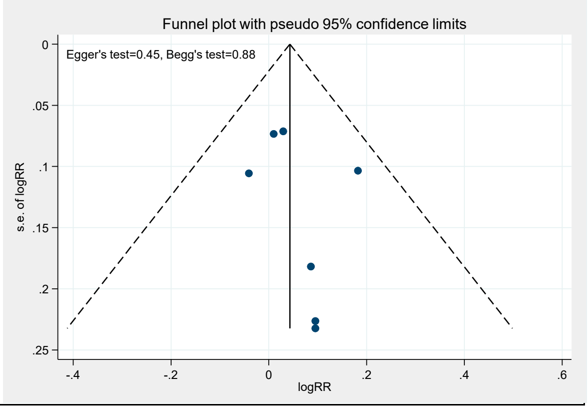

Supplement: Supplementary file 1 [file healthcare-09-00035-s001.pdf]
